# Damxungmacin A and B, Two New Amicoumacins with Rare Heterocyclic Cores Isolated from *Bacillus subtilis* XZ-7

**DOI:** 10.3390/molecules21111601

**Published:** 2016-11-23

**Authors:** Hui-Ling Tang, Cheng-Hang Sun, Xin-Xin Hu, Xue-Fu You, Min Wang, Shao-Wei Liu

**Affiliations:** 1 School of Life Science and Technology, China Pharmaceutical University, Nanjing 210009, Jiangsu, China; tanghuilingyaya@126.com; 2 Institute of Medicinal Biotechnology, Chinese Academy of Medical Sciences and Peking Union Medical College, Beijing 100050, China; chenghangsun@hotmail.com (C.-H.S.); huxinxin1985@163.com (X.-X.H.); xuefuyou@hotmail.com (X.-F.Y.); 3 School of Pharmacy, Jiangsu Food & Pharmaceutical Science College, Huaian 223005, Jiangsu, China

**Keywords:** Damxungmacin, amicoumacins, *Bacillus subtilis*, antibacterial activity, cytotoxic activity

## Abstract

Two new amicoumacins, named Damxungmacin A (**1**) and B (**2**), were isolated from the culture broth of a soil-derived bacterium *Bacillus subtilis* XZ-7. Their chemical structures were elucidated by spectroscopic studies (UV, IR, NMR and HR-ESI-MS). Compound **1** possessed a 1,4-diazabicyclo[2.2.1]heptane-2-one ring system in its structure, which was reported for the first time, while **2** had a 1-acetylmorpholine-3-one moiety, which was naturally rare. Compound **1** exhibited moderate to weak cytotoxic activities against three human tumor cell lines (A549, HCT116 and HepG2) with IC_50_ values of 13.33, 14.34 and 13.64 μM, respectively. Meanwhile, compound **1** showed weak antibacterial activities against some strains of *Staphylococcus epidermidis*, while compound **2** at 16 μg/mL did not show antibacterial activity.

## 1. Introduction

**Figure 1:**
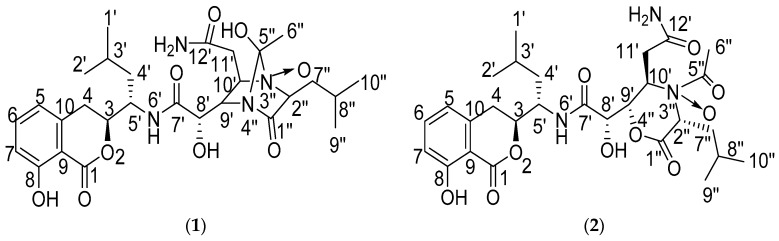
Structures of Damxungmacin A (**1**) and B (**2**).

Amicoumacins are a small group of isocoumarin derivatives produced by microorganisms. These compounds possess 3,4-dihydro-8-hydroxy-isocoumarin as the common skeleton and the structural variations are mainly attributed to the differences of the amide side chain [1]. They have been reported to exhibit a range of interesting bioactivities, such as antibacterial [2,3,4], cytotoxic [4,5,6,7], antiulcer [8,9,10], anti-inflammatory [11] and plant-growth inhibitory effects [12], which evoke great interest in medicinal research. In our ongoing research on novel amicoumacin products from *Bacillus* spp. [13,14], the strain *Bacillus subtilis* XZ-7, isolated from a soil sample collected from Damxung county in Tibet, China, was chosen through the chemical screening approaches and dereplication strategies [15]. Previous investigations of the strain XZ-7 have resulted in the identification and isolation of a series of amicoumacin compounds [15,16]. Recently, in our continuous study of novel metabolites from the strain, two new amicoumacins, Damxungmacin A (**1**) and B (**2**) (Figure 1), were isolated from its fermentation broth. Compound **1** possesses a 1,4-diazabicyclo[2.2.1]heptane-2-one ring system in its structure, which is reported for the first time, and **2** has a 1-acetylmorpholine-3-one moiety, which is naturally rare. Herein, we report the isolation, structural elucidation and in vitro antibacterial activity evaluation of **1** and **2**, together with the cytotoxicity evaluation of **1**.

## 2. Results and Discussion

### 2.1. Structure Elucidation of the Compounds

**Figure 2:**
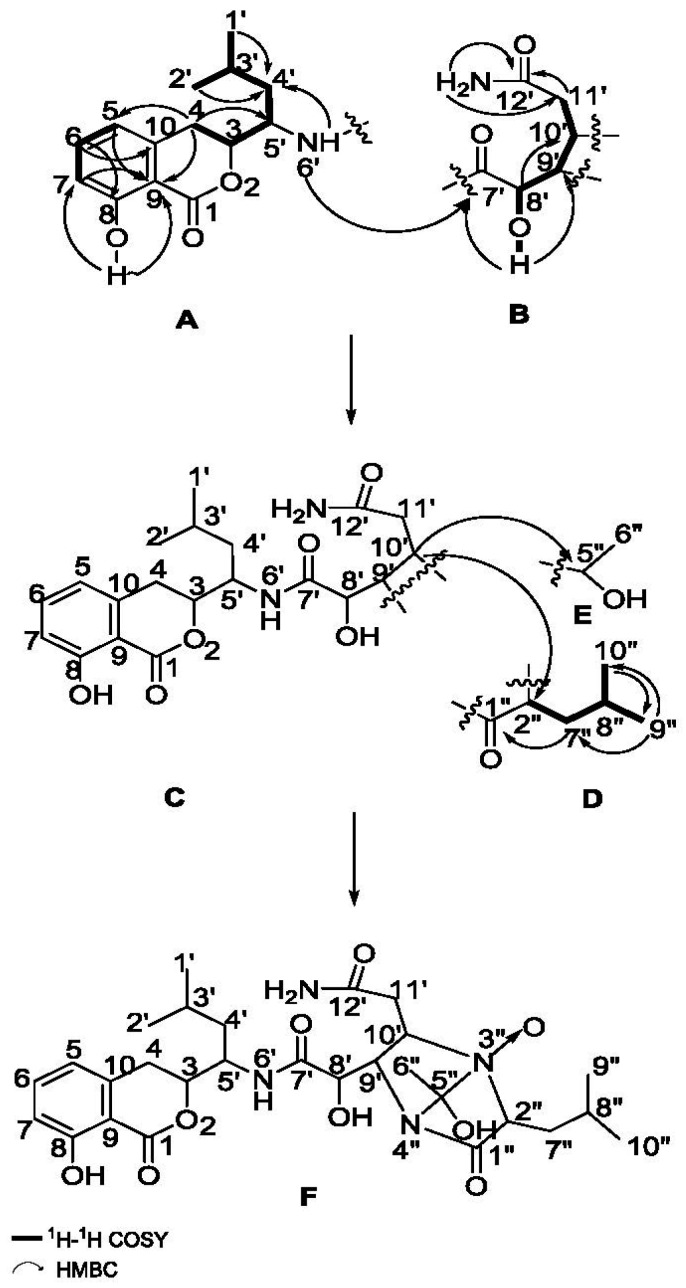
Partial structure elucidation and key ^1^H-^1^H COSY, HMBC correlations of **1**.

Compound **1** was isolated as a white amorphous powder. The molecular formula of C_28_H_40_N_4_O_9_ was determined by HR-ESI-MS at *m*/*z* 575.2723 [M − H]^−^ (calcd: 575.2722), indicating 11 degrees of unsaturation. The IR absorptions at 3350, 2958, 1669, 1462, 1231, 806 and 698 cm^−1^ indicated the presence of a benzoic acid moiety with a phenolic hydroxyl group and an amide group. The UV absorption at $\lambda_{\max}^{\mathrm{MeOH}}$ nm (log ε) 203 (4.43), 246 (3.74) and 314 (3.54) was nearly identical to those reported isocoumarins with characteristic UV spectral features [2,3,4,5,6,7,8,9,10,11,12,13,14,15,16]. The molecular formula suggested the presence of 28 carbon signals, but the ^13^C-NMR data (Table 1) displayed 26 carbon signals. Examination of the DEPT data indicated a methylene carbon signal (δ_C_ 40.1) embedded in DMSO-*d*_6_ solvent peaks, and a methyl carbon signal overlaid with a methylene carbon signal (δ_C_ 29.0). Four substructures (Figure 2), including the isocoumarin moiety (A), the functionalized adipate (B), the functionalized isocaproic acid (D) and the functionalized ethanol (E), were assigned by analyses of ^1^H-, ^13^C-, ^1^H-^1^H COSY, HSQC and HMBC NMR data recorded in DMSO-*d*_6_, as well as comparison with the literature data [2,3,4,5,6,7,8,9,10,11,12,13,14,15,16].

Elucidation of the part A started from the spin system, consisting of the three aromatic protons H-5 (δ_H_ 6.79, d, *J* = 7.2 Hz), H-6 (δ_H_ 7.45, t, *J* = 7.2 Hz) and H-7 (δ_H_ 6.81, d, *J* = 7.2 Hz), which displayed the coupling patterns for 1,2,3-trisubstituted benzenoid ring system. Their corresponding carbons were assigned by HSQC. The other three aromatic carbons were observed and assigned by tracing cross peaks in HMBC from H-5 and H-7 to C-9 (δ_C_ 108.2), and from H-6 to C-8 (δ_C_ 160.7) and C-10 (δ_C_ 141.3). The chemical shift of C-8 suggested a phenolic hydroxyl group should be attached to C-8, which was observed as a singlet proton signal in the downfield region of ^1^H-NMR (δ_H_ 10.82, s, 8-OH) for its hydrogen bond with the carbonyl group (1-C=O) [17,18]. HMBC correlations from 8-OH to C-7 and C-9 confirmed the connectivity between 8-OH and C-8. Another spin system was identified starting from two methyl groups, 1′-CH_3_ (δ_H_ 0.91, d, *J* = 6.0 Hz) and 2′-CH_3_ (δ_H_ 0.87, d, *J* = 6.0 Hz), both of which showed ^1^H-^1^H COSY correlations to H-3′ (δ_H_ 1.89, t, *J* = 6.0 Hz). The cross peaks of H-3′/H-4′ (δ_H_ 1.36; δ_H_ 1.66, t, *J* = 12.6 Hz) and H-4′/H-5′ (δ_H_ 4.19) in the ^1^H-^1^H COSY spectrum, together with the HMBC correlations from both H-1′ and H-2′ to C-4′ (δ_C_ 40.1) established the isopentyl group. The ^1^H-^1^H COSY correlations between H-5′/H-3 (δ_H_ 4.69, d, *J* = 12.6 Hz), between H-3/H-4 (δ_H_ 2.77, d, *J* = 16.8 Hz; *δ*_H_ 3.22, d, *J* = 12.6 Hz) together with the HMBC correlation from H-4 to C-5′ (δ_C_ 48.0) established the connectivity between C-5′ and C-3 (δ_C_ 81.4), C-3 and C-4 (δ_C_ 29.0). The HMBC correlations from H-4 to the aromatic carbons C-5 (δ_C_ 118.7) and C-9 indicated that C-4 was attached to C-10. The chemical shifts of H-3 and C-3 at low field indicated C-3 should be attached to the oxygen of the lactone ring to form the 3,4-dihydro-8-hydroxyisocoumarin. A cross peak could be observed between H-5′ and 6′-XH proton (δ_H_ 7.99, d, *J* = 3.0 Hz) in the ^1^H-^1^H COSY spectrum, and this 6′-XH proton was recognized as an NH proton on the basis of the chemical shifts of H-6′ and C-5′. All data above completed the construction of isocoumarin-type substructure A, which is the common structural moiety of all amicoumacins for their same biosynthetic pathway [19,20,21].

Substructure B was constructed starting from the methine proton H-8′ (δ_H_ 4.01) as a broad multiplet signal that showed a ^1^H-^1^H COSY correlation to H-9′ (δ_H_ 3.91). The fragment was expanded by ^1^H-^1^H COSY correlations of H-9′/H-10′ (δ_H_ 3.48), H-10′/H-11′ (δ_H_ 2.23, d, *J* = 11.4 Hz; δ_H_ 2.19, t, *J* = 12.0 Hz), together with the HMBC correlation from H-8′ to C-10′ (δ_C_ 64.9). The cross peak between H-8’ and 8’-OH (δ_H_ 5.52) in ^1^H-^1^H COSY spectrum, together with the HMBC correlations from 8′-OH to C-7′ (δ_C_ 171.7) and C-9′ (δ_C_ 76.4) suggested a hydroxyl group should be attached to C-8′. A carbon signal downfield at δ_C_ 172.6 in the ^13^C-NMR spectrum suggested the existence of 12′-C=O, which was connected to C-11′ (δ_C_ 36.4) by observation of the cross peak between H-11′ and C-12′ in the HMBC spectrum. Two singlet proton signals from 12′-NH_2_ (δ_H_ 6.87, s; δ_H_ 7.33, s) were readily observed in the downfield region of the ^1^H-NMR. HMBC correlations from 12′-NH_2_ protons to C-11′ and C-12′ suggested 12′-NH_2_ was attached to 12′-C=O. Thus, the substructure B as a functionalized adipate was established. Substructures A and B were connected on the basis of an HMBC correlation from the 6′-NH proton to C-7′, providing the substructure C.

Identification of the substructure D started from a carbon signal downfield at δ_C_ 167.8 in the ^13^C-NMR spectrum, which suggested the existence of 1″-C=O. The ^1^H-^1^H COSY correlations of H-2″ (δ_H_ 3.28)/H-7″ (δ_H_ 1.36, t, *J* = 6.0 Hz; δ_H_ 1.27, s), H-7″/H-8″ (δ_H_ 1.89, t, *J* = 6.0 Hz) together with HMBC correlations from H-2″ and H-7″ to C-1″, the structure moiety as –O=C(1″)–CH(2″)–CH_2_(7″)–CH(8″)–was established. Two methyl groups, 9″-CH_3_ (δ_H_ 0.82, d, *J* = 6.0 Hz) and 10″-CH_3_ (δ_H_ 0.79, d, *J* = 6.0 Hz), showed HMBC correlations to one another, and to C-7″ (δ_C_ 42.4), were both assigned to attached to C-8″ (δ_C_ 24.2). In addition, ^1^H-^1^H COSY correlations of H-8″/H-9″ and H-8″/H-10″ were readily observed. Therefore, the functionalized isocaproic acid, substructure D, was confirmed.

**Figure 3:**
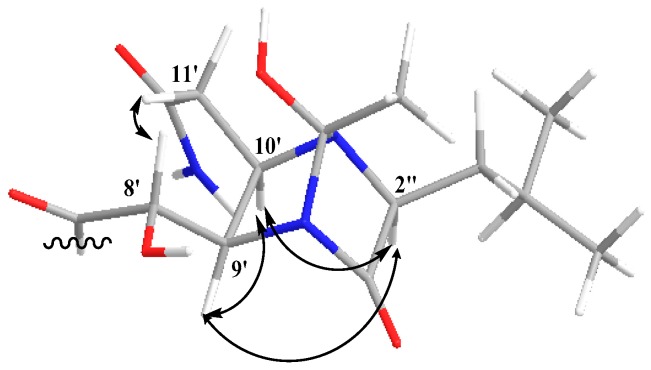
Key NOESY correlations of **1**.

**Figure 4:**
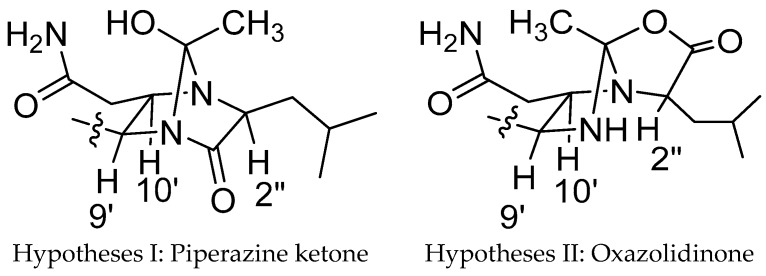
Possible hypotheses of connection ring of substructure C and D.

Substructures C and D accounted for nine degrees of unsaturation; therefore, the remaining two degrees of unsaturation were attributed to the existence of two cycle structures combining C with D. The carbon chemical shifts of C-9′, C-10′, C-2″ (δ_C_ 61.8) and C-5″ (δ_C_ 107.1) were indicative of bonding of an oxygen or nitrogen to these carbons. Cross peaks in the HMBC spectrum from H-10′ to C-2″ and C-5″ indicated C-2″ and C-5″ were linked with a nitrogen atom at C-10′. So, the junction of substructures C and E was determined. The NOESY correlation (Figure 3) between H-9′ and H-10′ confirmed that H-9′ and H-10′ were on the same side of the imidazoline ring. As there were no other signals in HMBC spectrum to confirm the means of connection of structure C and D, the exact connection may be between two possible hypotheses (Figure 4). By tracing the correlations from both H-9′ and H-10′ to H-2″ in the NOESY spectrum, the possible structure Ⅱ was ruled out, for the space distance between H-9′ and H-2″ in structure Ⅱ was too long to cause NOESY correlated signals. Overall, not only was a boat conformation of the structural moiety composed of 1″-C=O, 2″-CH, 3″-N, 4″-N, 9′-CH and 10′-CH established; but also the relative configuration of this substructure was elucidated.

Herein, all the other atoms in compound **1** were elucidated except an oxygen atom. Generally, this oxygen atom could exist as a peroxide bridge [22] or be co-coordinated by N and O donor ligands [23]. A peroxide bridge has seldom been presented in compounds that have several rich-electron groups; the nitrogen atoms with lone pair electrons are apt to form nitrogen-oxide [24]. The oxygen atom is inclined to tertiary amine N-3″ as its electron density is higher than acrylamide. All data above combined with the literature reports completed the elucidation of the structure of **1**.

**Figure 5:**
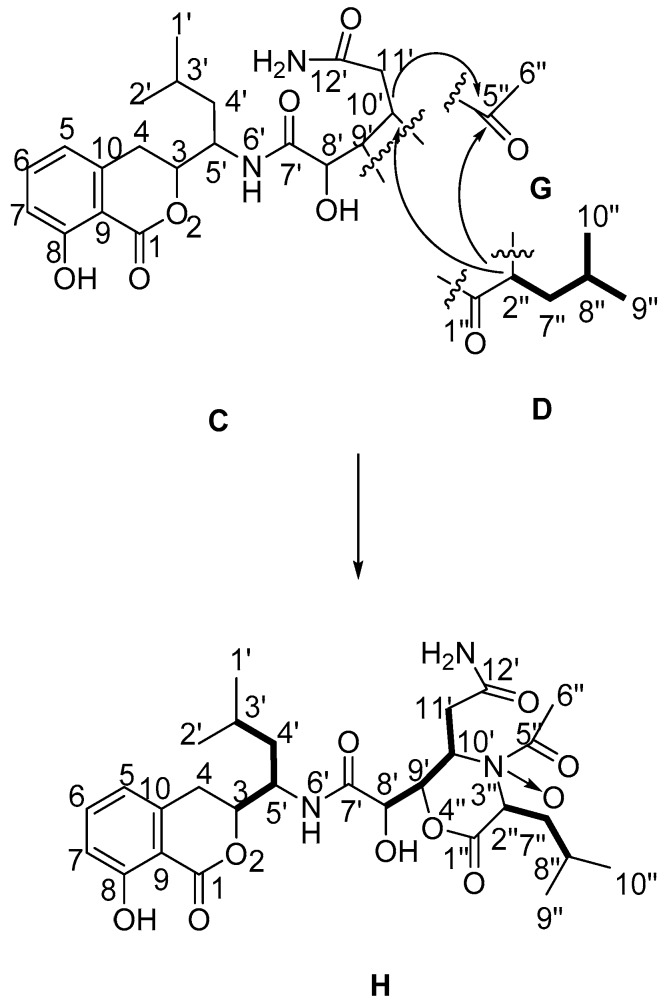
Key ^1^H-^1^H COSY and HMBC correlations of **2**.

Compound **2** was isolated as a white amorphous powder. The molecular formula of C_28_H_39_N_3_O_10_ was determined by HR-ESI-MS at *m*/*z* 576.2561 [M − H]^−^ (calcd: 576.2562), indicating 11 degrees of unsaturation. The UV absorption at 203, 246 and 314 nm indicated the presence of 3,4-dihydro-8-hydroxyisocoumarin skeleton. The molecular formula revealed the presence of 28 carbon signals, but the ^13^C-NMR data displayed 26 carbon signals, with another two methylene carbon signals (δ_C_ 39.5, δ_C_ 39.6) embedded in DMSO-*d*_6_ solvent peaks indicated by the DEPT spectrum. By comparison of the ^1^H- and ^13^C-NMR data of **2** with those of **1** (Table 1), substructures C and D could also be confirmed in compound **2**, but there was another substructure G (an acetyl group) presented in compound **2** rather than the substructure E in compound **1**. This finding was supported by the downfield carbon signal of C-5″ at δc 170.7, as well as the HMBC correlation from H-6″ (δ_H_ 2.07) to C-5″. By calculation of element composition and degrees of unsaturation of **2**, there could be a lactone ring to combine substructure C with D. The way of C-1″, C-2″ in substructure D linked to C-9′, C-10′ in substructure C also had two possibilities, scilicet, C-1″ may link to C-9′ or C-10′ while C-2″ links to the rest. The carbon chemical shifts of C-10′ (δ_C_ 51.6), C-2″ (δ_C_ 56.8) were indicative of a nitrogen bonding with these two carbons, and the cross peak between H-2″ (δ_H_ 4.04, t, *J* = 7.0 Hz) and C-10′ (δ_C_ 51.6) in the HMBC spectrum suggested C-10′ and C-2″ were linked with a nitrogen atom (N-3″) at C-10′. The HMBC correlations from H-2″ to C-5″, from H-10″ to C-5″ indicated the substructure G was attached to N-3″ that linked with C-2″ and C-10′. C-9′ (δ_C_ 85.3), whose carbon chemical shift was obviously higher than C-10′ and C-2″, was linked with an oxygen atom (O-4″) at C-1″. There was also an extra oxygen atom in **2** that linked with the nitrogen atom N-3″ to form a nitrogen-oxide coordination bond like **1**. Therefore, the structure of **2** was determined as in Figure 5.

By comparison of the NMR data of both compounds **1** and **2** with the literature [2,3,4,5,6,7,8,9,10,11,12,13,14,15,16], the relative configuration of isocoumarin moiety was readily confirmed (Figure 1). For the relative configuration of substructure acetylmorpholine in compound **2**, the Nuclear Overhauser Effect (NOE) was only observed at H-6″ when irritating H-2″, which suggested that the average distance between H-6″ and H-9′, H-10′ was considerably longer than between H-2″ and H-6″ and below the detection limit. Together with the fact that there was no signal-gain at H-10′ when irritating H-9′, the relative configuration of **2** was elucidated as in Figure 1. The ^1^H-NMR, ^13^C-NMR and HMBC data of **1** and **2** in DMSO-*d*_6_ were listed in Table 1.

### 2.2. Evaluation of Anti-Antibacterial Activities

In vitro antibacterial activities of compounds **1** and **2** were evaluated by the agar dilution method according to Clinic and Laboratory Standards Institute guidelines [25]. Compound **1** displayed inhibitory activities against some strains of *Staphylococcus* spp., including Methicillin-sensitive *Staphylococcus epidermidis* 13-1 (isolated from the clinic) with the MIC value of 16 μg/mL, Methicillin-sensitive *Staphylococcus epidermidis* ATCC 12228 with the MIC value of 32 μg/mL, Methicillin-sensitive *Staphylococcus aureus* ATCC 29213 and Methicillin-resistant *Staphylococcus aureus* ATCC 33591 with the MIC value of 64 μg/mL. For the inadequate amount, the maximum tested concentration of compound **2** was only 16 μg/mL, and compound **2** didn’t show any antibacterial activity at 16 μg/mL. The MIC values against bacteria of **1** and **2** are listed in Table S1 in the Supplementary Materials.

### 2.3. Anti-Proliferative Assay on Human Tumor Cells

Compound **1** was evaluated in vitro for its cytotoxic activity against three human tumor cell lines (human lung adenocarcinoma epithelial cells A549, human colon cancer cells HCT116 and human liver hepatocellular cells HepG2) using the MTT assay. Compound **1** showed moderate to weak cytotoxicity in vitro against A549, HCT116 and HepG2 cell lines, with IC_50_ values of 13.33, 14.34 and 13.64 μM, respectively.

## 3. Experimental Section

### 3.1. General Information

Optical rotations were obtained on a Perkin-Elmer 343 polarimeter (Perkin-Elmer, Waltham, MA, USA). UV spectra were measured on a Shimadzu UV-2550 spectrometer (Shimadzu Corp., Kyoto, Japan). The IR spectra were acquired with a Thermo Nicolet 5700 FT-IR spectrometer (Thermo Fisher Scientific, Waltham, MA, USA). HR-ESI-MS spectra were recorded on a Bruker solariX FT-ICR-MS (Bruker Daltonics Inc., Billerica, MA, USA). NMR spectra were recorded on Bruker Avance III 600 MHz NMR spectrometer (Bruker Biospin GmbH, Reinsteten, Germany) operating at 600 MHz for ^1^H-NMR and 150 MHz for ^13^C-NMR. Samples were dissolved in 0.6 mL DMSO-*d*_6_ solvent and transferred to 3 mm NMR tubes. Coupling constants were expressed in Hz and chemical shifts were presented as δ (ppm) values with tetramethylsilane (TMS) as the internal standard. The ^1^H-^1^H COSY, HSQC, HMBC and NOE NMR experiments were carried out using standard pulse sequences, and parameters of HSQC and HMBC were optimized for coupling constants of 145.0 and 8.0 Hz, respectively. Data processing was performed with the Topspin software (Bruker Biospin GmbH, Reinsteten, Germany). Reversed phase medium pressure liquid chromatography (RP-MPLC) was performed on a LiChroprep RP-C18 column (50 × 1.0 cm, 40–63 μm, Merck, Darmstadt, Germany). Semi-preparative HPLC was carried out on an HPLC system composed of LC-20AT pumps with an SPD-M20A detector (Shimadzu Corp.). The HPLC separation was firstly carried out on a Zorbax SB-C18 column (250 × 9.4 mm, 5 μm, Agilent Technologies Inc., Santa Clara, CA, USA), and further purified on a Zorbax Bonus-RP column (150 × 4.6 mm, 3.5 μm, Agilent Technologies Inc.). Fractions were monitored by the UPLC-DAD-MS system (LC-20AD, SPD-M20A and LCMS-2020, Shimadzu Corp).

### 3.2. Bacterial Material

The producing strain, *Bacillus subtilis* XZ-7 was isolated from a soil sample collected from Damxung County in Tibet, China. The strain XZ-7 was identified as *Bacillus subtilis* subsp. *Inaquosorum* according to its morphological characteristics and analysis of 16S rRNA sequence. A stock culture of the strain was maintained on modified ISP2 agar slants consisting of glucose 4.0 g, yeast extract 4.0 g, malt extract 5.0 g, thiamine 0.5 mg, riboflavin 0.5 mg, pantothenic acid 0.5 mg, pyridoxine 0.5 mg, inositol 0.5 mg, D-biotin 0.5 mg, nicotinic acid 0.5 mg, para-aminobengoic acid 0.5 mg, l-Phenylalanine 1.0 mg, d-alanine 0.3 mg, microelement solution 1.0 mL (FeSO_4_ 2.0 mg, MnCl_2_ 1.0 mg, ZnSO_4_·7H_2_O 1.0 mg) and agar 20.0 g in 1.0 L distilled water (pH 8.0).

### 3.3. Fermentation, Extraction and Isolation

Strain XZ-7, growing on the modified ISP2 agar slant at 28 °C for 3 days, was inoculated into 500 mL Erlenmeyer flasks containing 100 mL of seed medium (modified ISP2 liquid medium). The flasks were shaken at 180 r.p.m at 28 °C on rotary shakers for 24 h to obtain seed cultures, then the seed cultures were transferred to 5000 mL Erlenmeyer flasks containing 1000 mL of the modified ISP2 liquid medium to scale up the cultures. After incubation at 180 r.p.m at 28 °C for 24 h, the fermentation broth (200 L) was centrifuged and then the supernatant was extracted with an equal volume of ethyl acetate (EtOAc) at room temperature. The organic layer was concentrated by rotary evaporation to yield a crude extract of brown syrup (3.2 g).

The concentrated extract was subjected to RP-MPLC on a LiChroprep RP-C_18_ column and eluted successively with 30%, 60% and 90% aqueous methanol (each 200 mL). Fractions were monitored by the UPLC-DAD-MS system, and the fractions containing different molecular weights of known amicoumacins were combined and concentrated under vacuum to give a yellow syrup (204.1 mg). After being dissolved in 1.0 mL of methanol, the sample was subjected to semi-preparative HPLC on Zorbax SB-C_18_ column with methanol/water 61:39 (*v*/*v*) at 1 min·mL^−1^. Fractions with UV absorption maxima at 203, 246 and 314 nm were further purified by HPLC on a Zorbax Bonus-RP column with methanol/water 53:47 (*v*/*v*) at 1 min·mL^−1^ to obtain compound **1** (7.9 mg) and compound **2** (1.9 mg).

### 3.4. Spectroscopic Data of Compounds

Damxungmacin A (**1**): white powder; [α]${}_{D}^{20}$ = −66.0 (*c* 0.60, MeOH); UV (MeOH) λ_max_ nm (log *ε*): 203 (4.43), 246 (3.74), 314 (3.54); IR (KBr) ν_max_ 3350, 2958, 2870, 1669, 1537, 1462, 1231, 1112, 1040, 806 and 698 cm^−1^; ^1^H- and ^13^C-NMR data, see Table 1; HR-ESI-MS: *m*/*z* 575.2723 [M − H]^−^ (calcd for C_28_H_40_N_4_O_9_, 575.2722).

Damxungmacin B (**2**): white powder; [α]${}_{D}^{20}$ = −32.0 (*c* 0.55, MeOH); UV (MeOH) λ_max_ nm (log *ε*): 203 (4.51), 246 (3.80), 314 (3.64); IR (KBr) ν_max_ 3320, 2957, 2921, 2850, 1775, 1668, 1530, 1463, 1202, 1112, 1040, 807 and 698 cm^−1^; ^1^H- and ^13^C-NMR data, see Table 1; HR-ESI-MS: *m*/*z* 576.2561 [M − H]^−^ (calcd for C_28_H_39_N_3_O_10_, 576.2562).

### 3.5. Cytotoxicity Assay In Vitro

The in vitro cytotoxic activity of **1** was tested according to the MTT method [26] using three human tumor cell lines: A549, HCT116 and HepG2. The cells were obtained commercially from the American Type Culture Collection (ATCC, Rockville, MD, USA). In brief, compound **1** was dissolved in DMSO to make the stock solution, then diluted in RPMI-1640 culture medium (Gibco BRL Corp., Gaithersburg, MD, USA) at different concentrations. 100 μL cell suspension containing about 3 × 10^3^ cells were seeded into wells of 96 well plates. After overnight incubation, different concentrations of **1** were added, and the final concentrations were 2.5, 5, 10, 20, 40, 80, 100 μM, respectively, and then incubated for 48 h. Afterwards, the supernatant was removed and 20 μL MTT were added to each well and incubated for 20 min. The absorbance at 570 nm of each well was monitored by a Multiskan MK3 microplate reader (Thermo Labsystems, Vantaa, Finland).

### 3.6. Antibacterial Assay In Vitro

The antibacterial activities of compounds **1** and **2** were evaluated by agar dilution method. Bacteria strains used in this study were obtained from Department of Pharmacology, Institute of Medicinal Biotechnology. **1** and **2** were dissolved in DMSO and two-fold serially diluted in Mueller-Hinton broth. The final concentrations of compounds ranged from 0.125 to 64 μg/mL for **1**, and from 0.125 to 16 μg/mL for **2**. Bacterial strains tested were incubated in Mueller-Hinton broth for 12 h and then diluted to 0.5 McFarland standard. The bacteria tested were inoculated on Mueller-Hinton agar plates after 10 fold dilution by the MIT-P multipoint inoculator (Sakuma, Tokyo, Japan). After incubation at 37 °C for 16–24 h, the MICs were recorded.

## 4. Conclusions

Two novel amicoumacins, named Damxungmacin A (**1**) and B (**2**), were detected by UPLC-DAD-MS system, extracted by ethyl acetate and purified by various chromatographies from the fermentation broth of the bacterium *Bacillus subtilis* XZ-7. The structures of the new compounds were identified based on extensive spectroscopic analysis and published analogues. The structures of Damxungmacins are unique for their nitrogen-containing heterocyclic cores. The 1,4-diazabicyclo[2.2.1]heptane-2-one ring system in **1** is unprecedented both in natural and synthetic compounds. The 1-acetylmorpholine-3-one moiety in **2** is rare in nature products. Compound **1** exhibited moderate to weak cytotoxic activities against three human tumor cell lines (A549, HCT116 and HepG2), and displayed weak antibacterial activities against some strains of *Staphylococcus epidermidis*. For compound **2**, it did not exhibit inhibitory activity against all tested strains at 16 μg/mL.

## Figures and Tables

**Table 1:** ^1^H-, ^13^C-NMR and HMBC data for compounds **1** and **2** in DMSO-*d*_6_.

No.	Damxungmacin A (1)			Damxungmacin B (2)		
	δ_C_^a^	δ_H_ (mult, *J* in Hz) ^b^	HMBC	δ_C_^a^	δ_H_ (mult, *J* in Hz) ^b^	HMBC
1	169.4			168.9		
3	81.4	4.69 (1H, d, *J* = 12.6 Hz)	10	80.6	4.68 (1H, d, *J* = 8.4 Hz)	
4	29.0	3.22 (1H, t, *J* = 12.6 Hz) 2.77 (1H, d, *J* = 16.8 Hz)	3,5,9,10,5′	29.1	2.99 (1H, t, *J* = 12.6 Hz) 2.90 (1H, d, *J* = 12.6 Hz)	3,9,10
5	118.7	6.79 (1H, d, *J* = 7.2 Hz)	4,7,8,9	118.4	6.82 (2H, unresolved multiplet)	
6	136.2	7.45 (1H, t, *J* = 7.2 Hz)	7,8,9,10	136.3	7.48 (1H, unresolved multiplet)	
7	114.9	6.81 (1H, d, *J* = 7.2 Hz)	1,5,6,8,9,10	115.4	6.84 (2H, unresolved multiplet)	
8	160.7			161.0		
8-OH		10.82 (1H, s)	7,8,9		Unidentified	
9	108.2			108.4		
10	141.3			140.5		
1′	25.0	0.91 (3H, d, *J* = 6.0 Hz)	3′,4′	22.7	0.91 (3H, d, *J* = 6.0 Hz)	2′,3′,4′
2′	21.7	0.87 (3H, d, *J* = 6.0 Hz)	3′,4′	22.6	0.89 (3H, d, *J* = 6.0 Hz,)	1′,3′,4′
3′	23.1	1.89 (1H, t, *J* = 6.0 Hz)	4′	23.8	1.43(1H, m)	1′,2′,4′
4′	40.1	1.66 (1H, t, *J* = 12.6 Hz) 1.36 (1H, unresolved multiplet)	1′,2′,3′	39.5	1.66 (1H, m, overlap) 1.36 (1H, m, overlap)	3,1′,2′,3′,5′
5′	48.0	4.19 (1H, unresolved multiplet)	4′,7′	48.2	4.17 (1H, m)	3′,4′,7′
6′-NH		7.99 (1H, d, *J* = 3.0 Hz)	4′,5′,7′,8′		7.67 (1H, d, *J* = 9.6 Hz)	5′,7′
7′	171.7			170.6		
8′	68.9	4.01 (1H, unresolved multiplet)	7′,9′,10′	72.01	4.12 (1H, d, *J* = 3.0 Hz)	7′,9′
8′-OH		5.52 (1H, unresolved multiplet))	7′,8′,9′		Unidentified	
9′	76.4	3.91 (1H,unresolved multiplet)	7′,8′,11′	85.3	4.39 (1H, t, *J* = 3.0 Hz)	7′,8′,10′,12′
10′	64.9	3.48 (1H, unresolved multiplet)	12′,2″,5″	51.6	4.54(1H, dt, *J* = 10.2 Hz)	5″,8′,9′,11′,12′
11′	36.4	2.23 (1H, d, *J* = 11.4 Hz) 2.19 (1H, t, *J* = 12.0 Hz)	9′,10′,12′	34.9	2.61 (1H, dd, *J* = 10.2, 18.0 Hz) 2.18 (1H, dd, *J* = 10.2, 18.0 Hz)	9′,10′,12′
12′	172.6			175.5		
12′-NH_2_		7.33 (1H, s) 6.87 (1H, s)	11′,12′		Unidentified	
1″	167.8			166.2		
2″	61.8	3.28 (1H, unresolved multiplet)	10′,3″,7″	56.8	4.04 (1H, t, *J* = 7.0 Hz)	10′,1″,5″,7″,8″
5″	107.1			170.7		
5″-OH		9.66 (1H, s)			Unidentified	
6″	29.0	1.28 (3H, s)		23.3	2.07 (3H, s)	2″,5″
7″	42.4	1.36 (1H, t, *J* = 6.0 Hz) 1.27 (1H, s, overlap)	2″,3″,8″	39.6	1.45 (1H, m) 1.35 (1H, m)	2″,8″,9″
8″	24.2	1.89 (1H, t, *J* = 6.0 Hz)	2″,7″,9″,10″	24.2	1.44 (1H, m, overlap)	2″,7″,9″,10″
9″	22.7	0.82 (3H, d, *J* = 6.0 Hz)	7″,8″,10″	22.3	0.890 (3H, d, *J* = 6.6 Hz)	8″,10″
10″	23.4	0.79 (3H, d, *J* = 6.0 Hz)	7″,8″,9″	21.6	0.825 (3H, d, *J* = 6.6 Hz)	8″,9″

^a^ Recorded at 150 MHz; ^b^ Recorded at 600 MHz.

## Supplementary Materials

The following are available online at: http://www.mdpi.com/1420-3049/21/11/1601/s1, HR-ESI-MS and NMR spectra data of Damxungmacin A and B as Supplementary Materials.

## Abbreviations

The following abbreviations are used in this manuscript:

UV	Ultraviolet
IR	Infrared
NMR	Nuclear magnetic resonance
HR-ESI-MS	High resolution electrospray ionization mass spectroscopy
RP-MPLC	Reverse-phase medium-oressure liquid chromatography
UPLC-DAD-MS	Ultra-performance liquid chromatography coupled with diode array detection and mass spectroscopy
