# Supplementary material for: Damxungmacin A and B, Two New Amicoumacins with Rare Heterocyclic Cores Isolated from Bacillus subtilis XZ-7
Source: Molecules. 2016 Nov 23;21(11):1601. doi: 10.3390/molecules21111601 (PMC6274204; doi:10.3390/molecules21111601)
Supplement: Supplementary file 1 [file molecules-21-01601-s001.pdf]

Supplementary Materials: Damxungmacin A and B, Two New Amicoumacins with Rare Heterocyclic Cores Isolated from *Bacillus subtilis* XZ-7

Hui-Ling Tang, Cheng-Hang Sun, Xin-Xin Hu, Xue-Fu You, Min Wang and Shao-Wei Liu

Table S1. Antibacterial activities of **1** and **2**.

Test Organisms	Test Organism No.	Drug Resistance	MIC (μg/mL)	
			**1**	**2**
Staphylococcus epidermidis	ATCC 12228	MSSE	32	>16
Staphylococcus epidermidis	13-1 *	MSSE	16	>16
Staphylococcus epidermidis	13-3 *	MRSE	>64	>16
Staphylococcus aureus	ATCC 29213	MSSA	64	>16
Staphylococcus aureus	ATCC 33591	MRSA	64	>16
Staphylococcus aureus	13-17 *	MSSA	>64	>16
Staphylococcus aureus	13-18 *	MRSA	>64	>16
Enterococcus faecalis	ATCC 29212	VSE	>64	>16
Enterococcus faecalis	ATCC 51299	VRE	>64	>16
Enterococcus faecalis	13-4 *	VSE	>64	>16
Enterococcus faecium	ATCC 700221	VRE	>64	>16
Escherichia coli	ATCC 25922	ESBLs (−)	>64	>16
Escherichia coli	1515 *	ESBLs (−)	>64	>16
Escherichia coli	14-11 *	ESBLs (+)	>64	>16
Klebsiella pneumoniae	ATCC 700603	ESBLs (+)	>64	>16
Klebsiella pneumoniae	7 *	ESBLs (−)	>64	>16
Klebsiella pneumoniae	ATCC BAA-2146	NDM-1 (+)	>64	>16
Pseudomonas aeruginosa	PAO1		>64	>16
Pseudomonas aeruginosa	13-46 *		>64	>16
Acinetobacter calcoacetious	ATCC 19606		>64	>16
Enterobacter cloacae	ATCC 43560		>64	>16
Enterobacter aerogenes	ATCC 13048		>64	>16
Serratia marcescens	ATCC 21074		>64	>16
Morganella morganii	ATCC 25830		>64	>16
Providentia rettgeri	ATCC 31052		>64	>16
Proteus vulgaris	ATCC 29905		>64	>16
Proteus mirabilis	13-1 *		>64	>16
Stenotrophomonas maltophilia	ATCC 13636		>64	>16
Citrobacter freundii	ATCC 43864		>64	>16

MSSE: Methicillin-sensitive *Staphylococcus epidermidis*; MRSE: Methicillin-resistant *Staphylococcus epidermidis*; MSSA: Methicillin-sensitive *Staphylococcus aureus*; MRSA: Methicillin-resistant *Staphylococcus aureus*; VSE: Vancomycin-sensitive *Enterococci*; VRE: Vancomycin-resistant *Enterococci*; ESBLs (+)/(−): with/without Extended-Spectrum Beta-Lactamases; NDM-1 (+): with New Delhi metallo-β-lactamase 1; *: Isolated from the clinic.

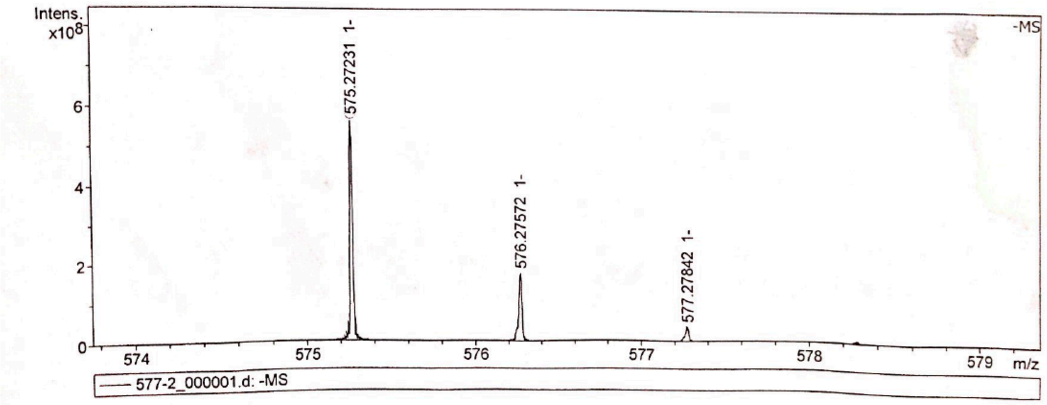

Meas. m/z	#	Ion Formula	Score	m/z	err [ppm]	Mean err [ppm]	mSigma	rdb	e⁻ Conf	N-Rule
339.233022	1	C23H31O2	100.00	339.232954	-0.2	-0.2	6.6	8.5	even	ok
405.166890	1	C20H25N2O7	100.00	405.166725	0.4	-0.6	9.4	9.5	even	ok
	2	C19H19N9O2	67.64	405.166719	0.4	-0.9	14.7	15.0	odd	ok
475.208894	1	C24H31N2O8	100.00	475.208590	-0.6	-0.8	10.2	10.5	even	ok
	2	C23H25N9O3	92.65	475.208584	-0.7	-1.1	14.1	16.0	odd	ok
575.272310	1	C28H39N4O9	100.00	575.272252	0.1	-0.2	9.9	11.5	even	ok
611.249295	1	C28H40ClN4O9	100.00	611.248930	0.6	-0.0	37.8	10.5	even	ok
	2	C29H36ClN8O5	59.11	611.250268	-1.6	2.0	45.5	15.5	even	ok

Figure S1. HR-ESI-MS of Damxungmacin A (**1**).

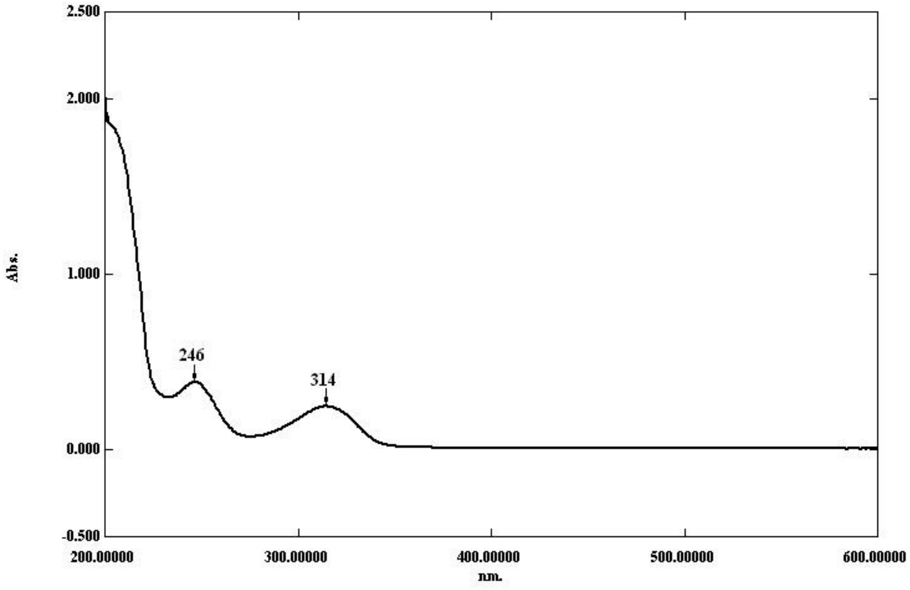

Figure S2. UV spectrum of Damxungmacin A (**1**).

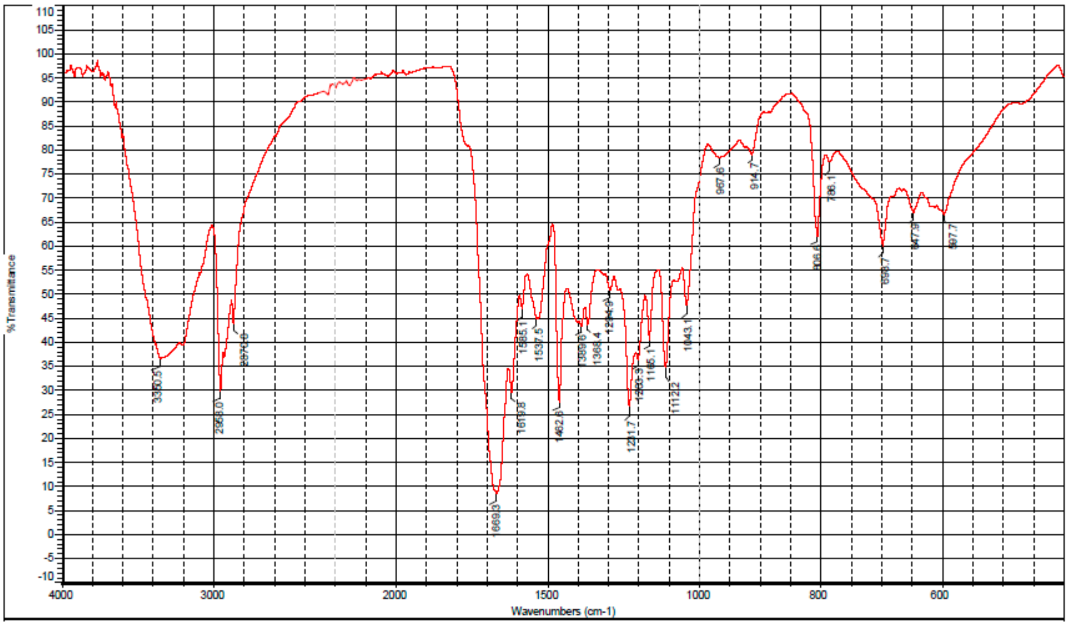

Figure S3. IR spectrum of Damxungmacin A (**1**).

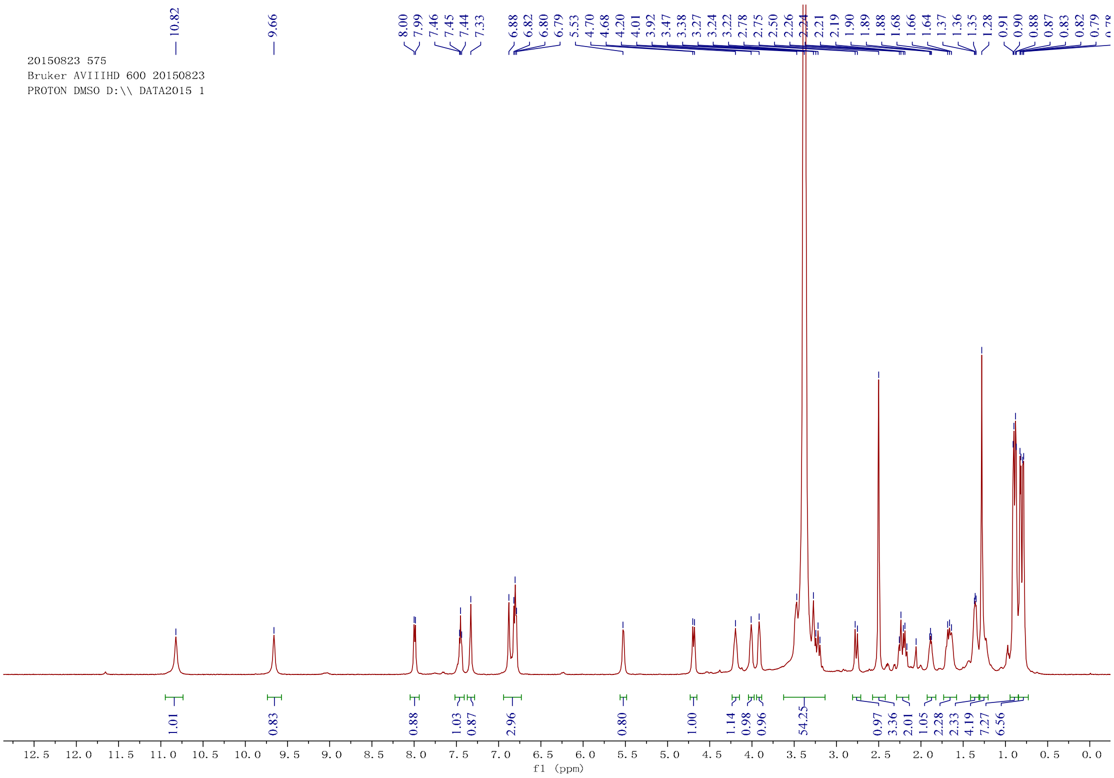

Figure S4. ^{1}H-NMR spectrum of Damxungmacin A (**1**).

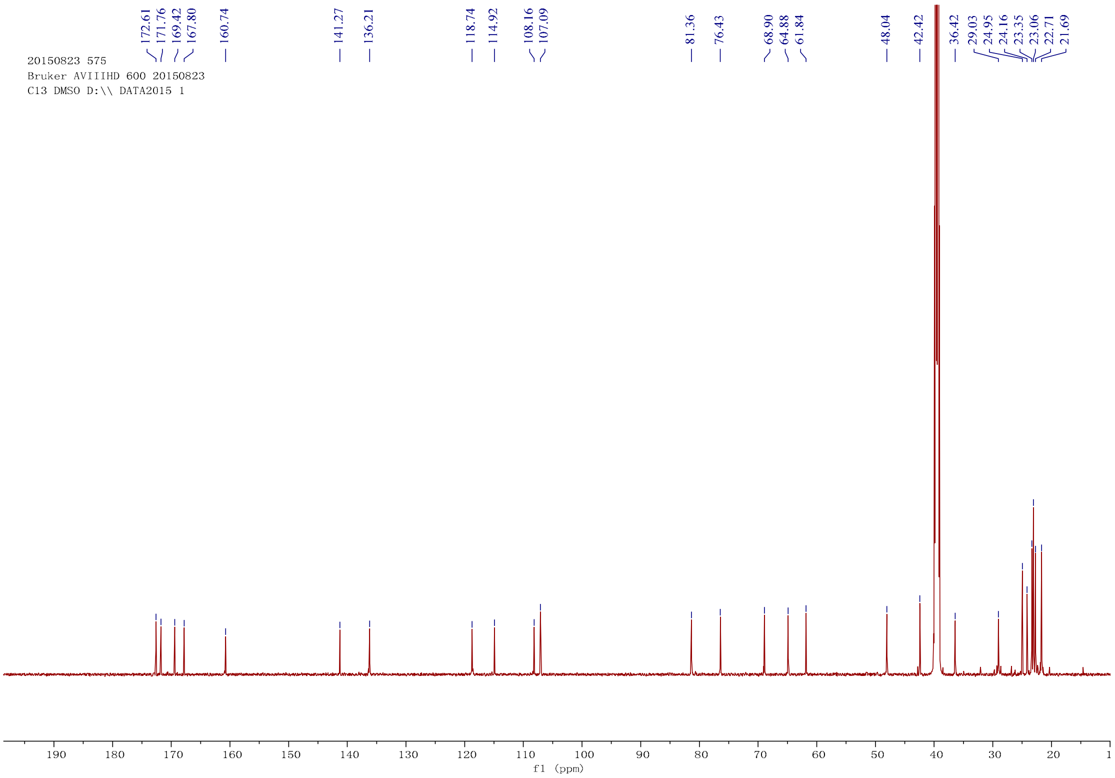

Figure S5. ^{13}C-NMR spectrum of Damxungmacin A (**1**).

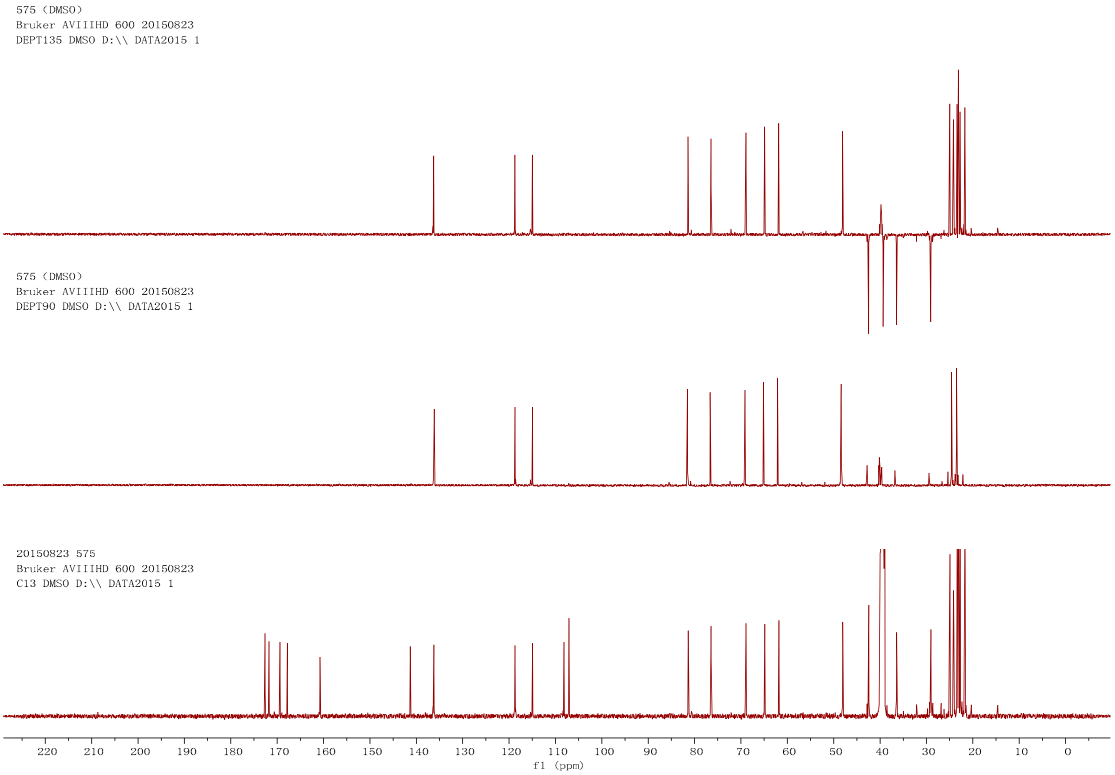

Figure S6. DEPT spectrum of Damxungmacin A (**1**).

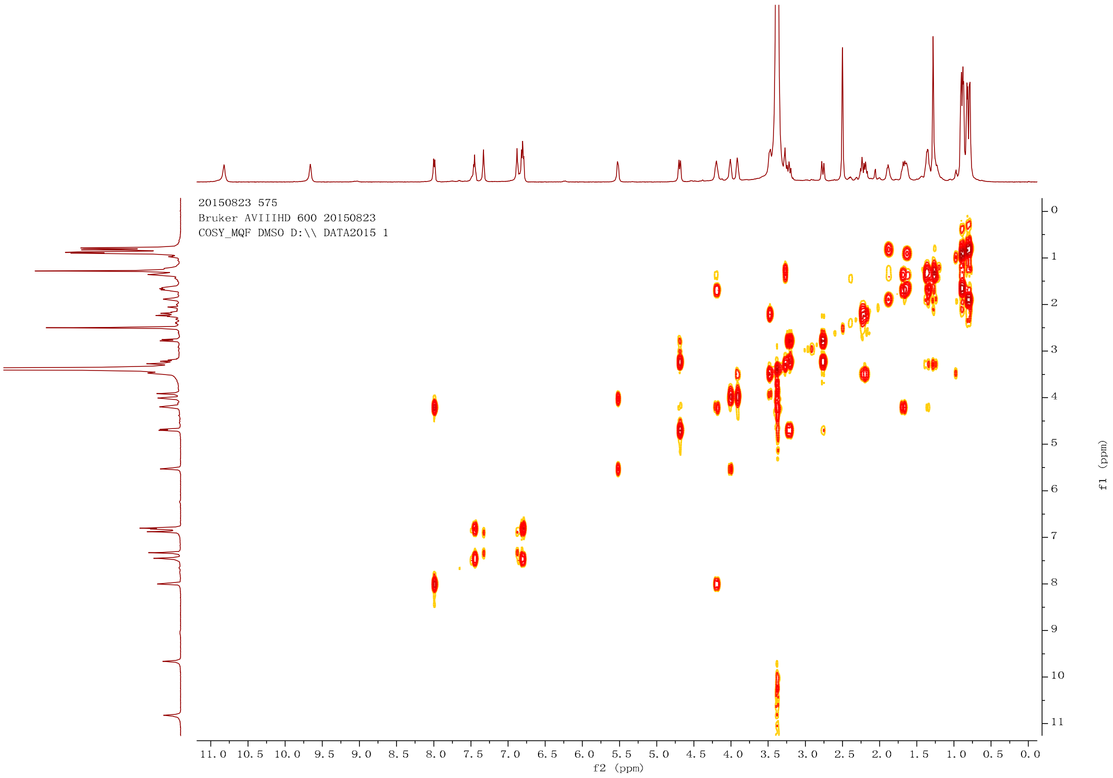

Figure S7. ^{1}H-^{1}H COSY spectrum of Damxungmacin A (**1**).

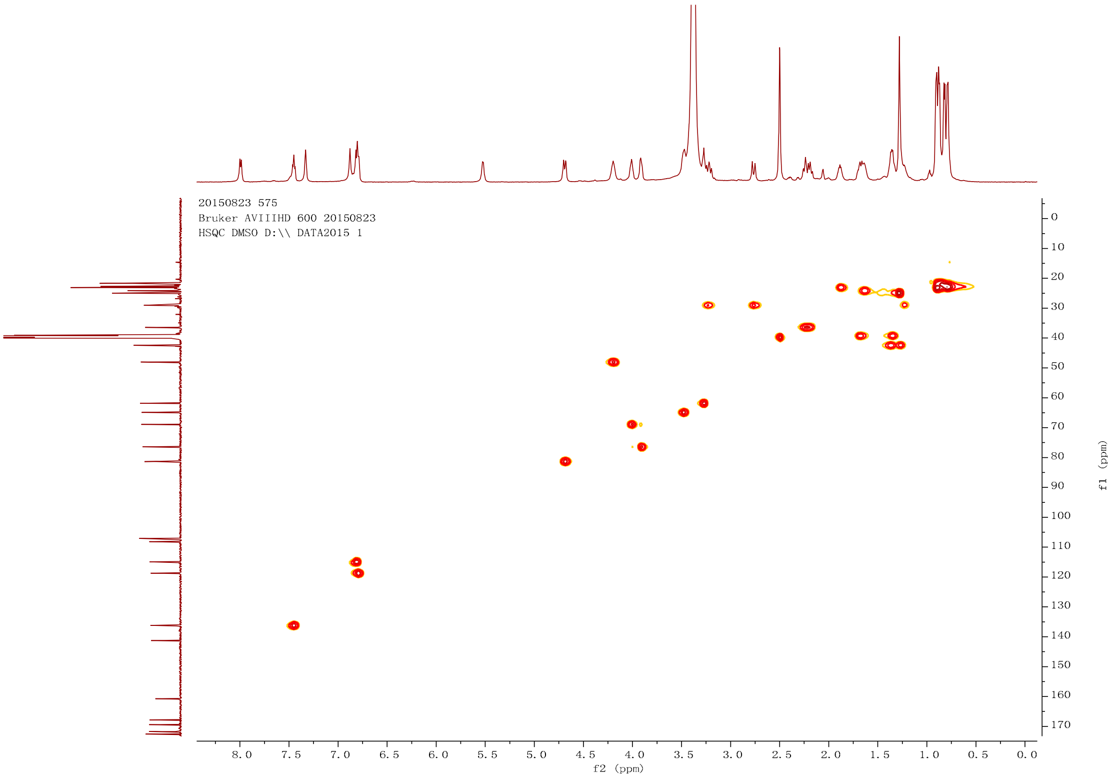

Figure S8. HSQC spectrum of Damxungmacin A (**1**).

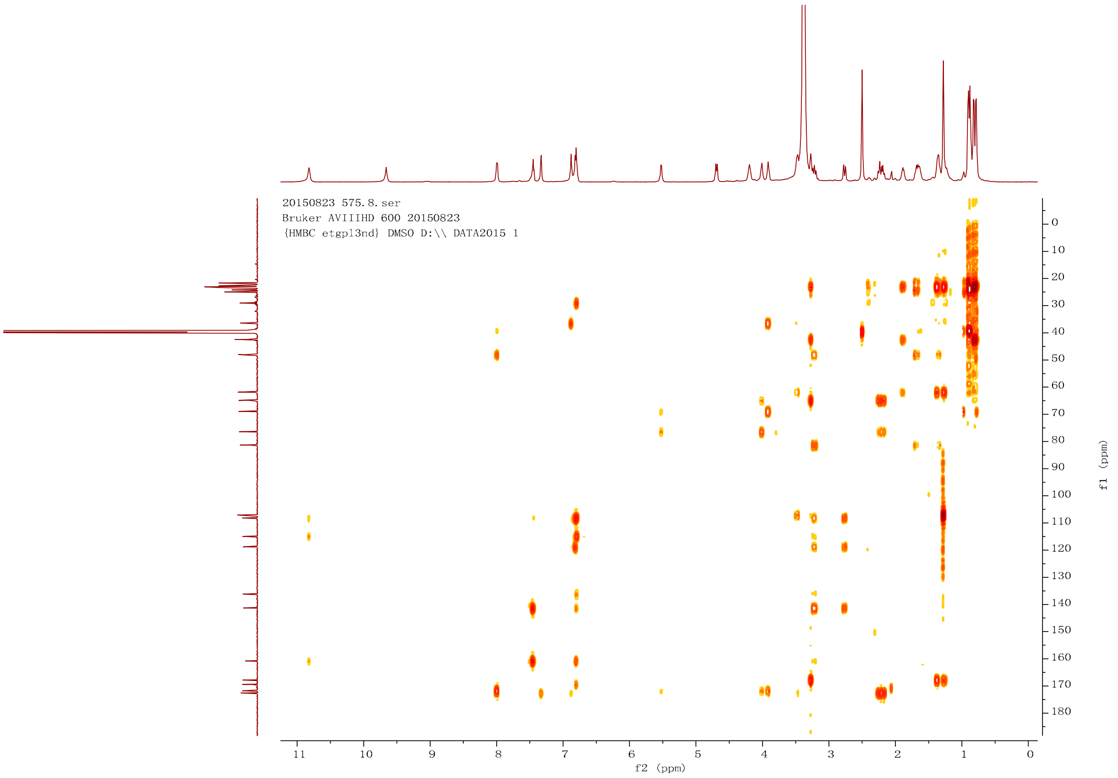

Figure S9. HMBC spectrum of Damxungmacin A (**1**).

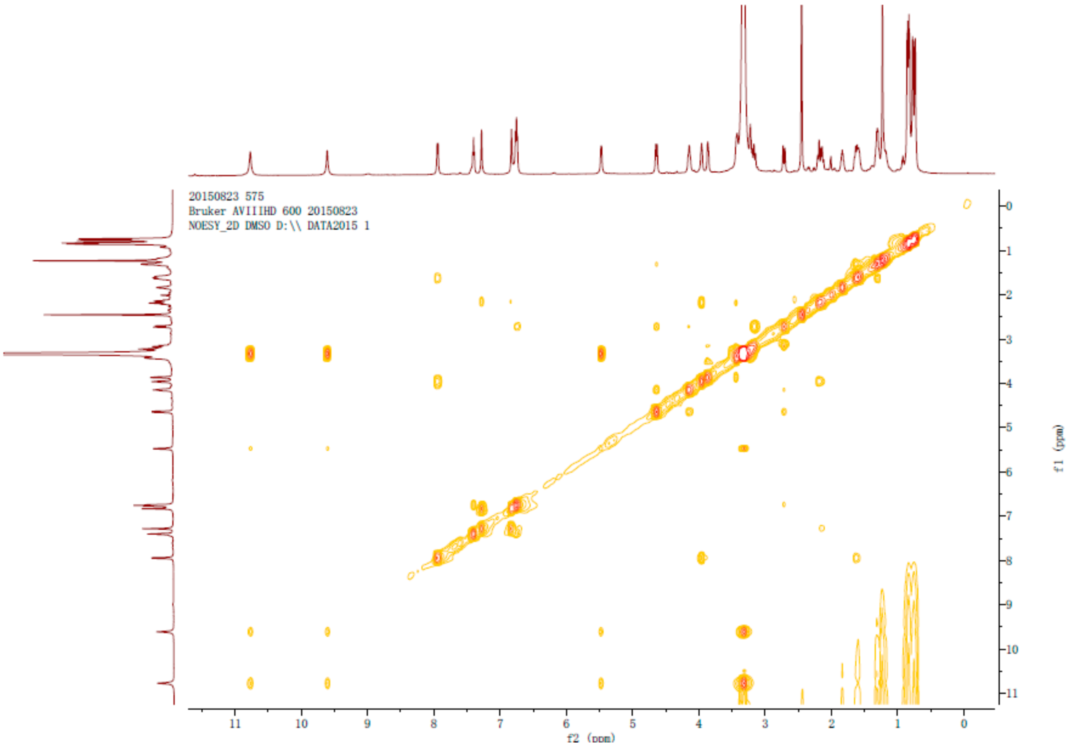

Figure S10. 2D NOESY spectrum of Damxungmacin A (**1**).

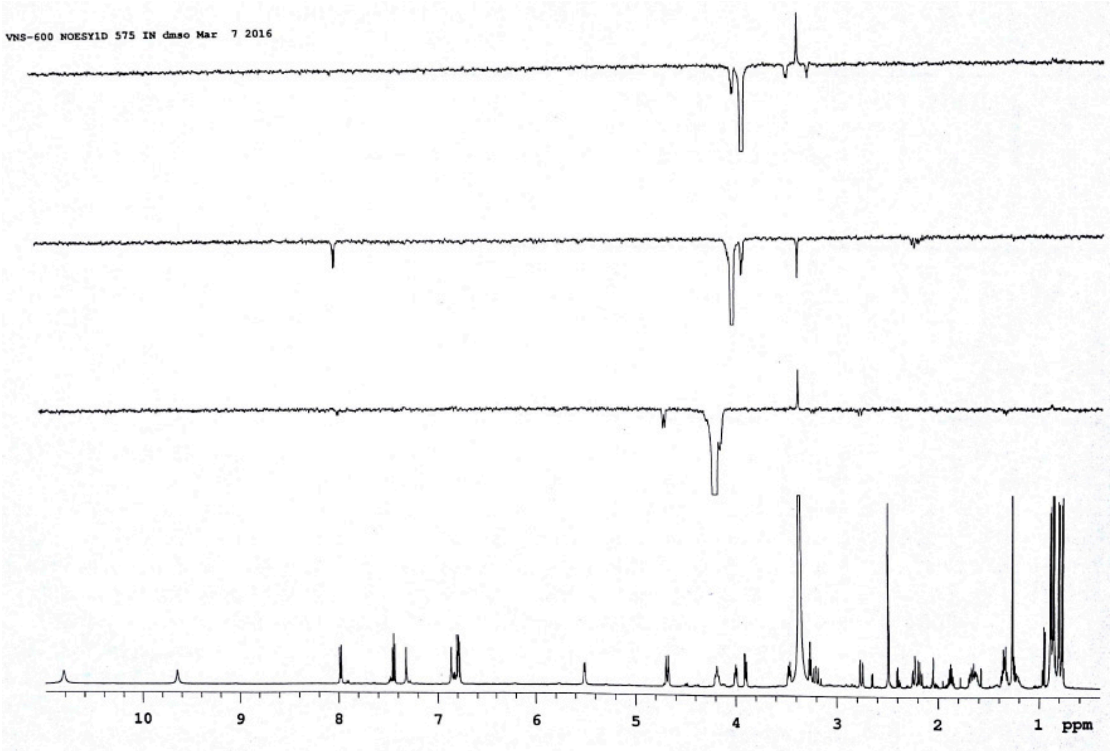

Figure S11. 1D NOESY spectrum of Damxungmacin A (**1**).

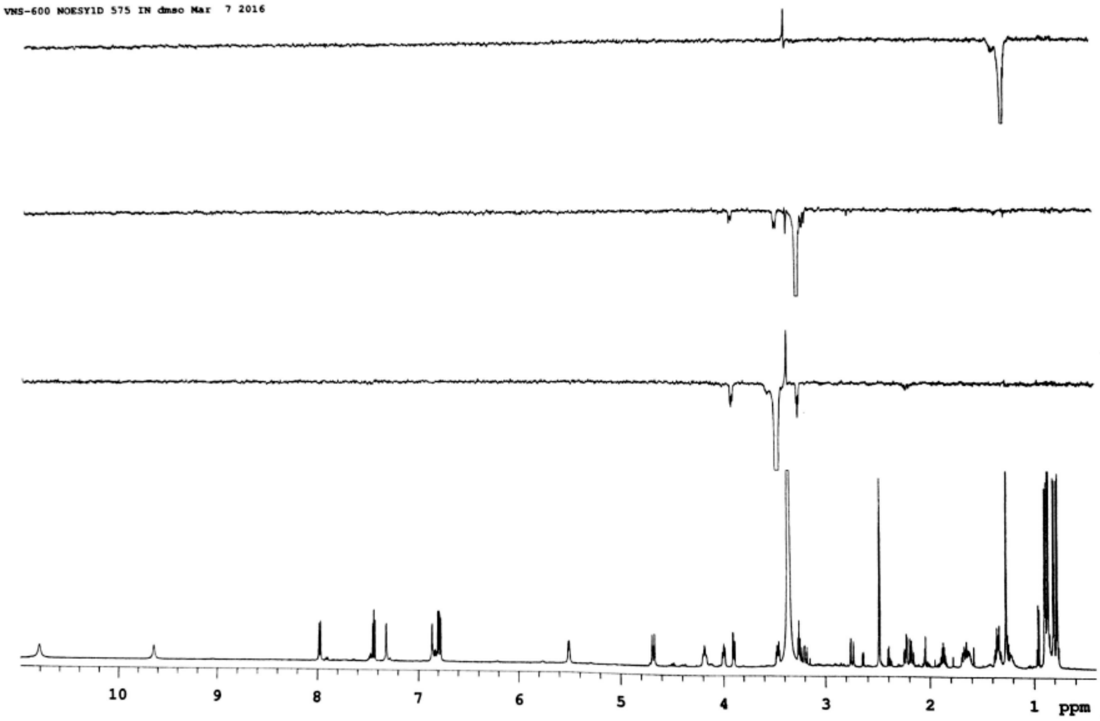

Figure S12. 1D NOESY spectrum of Damxungmacin A (**1**).

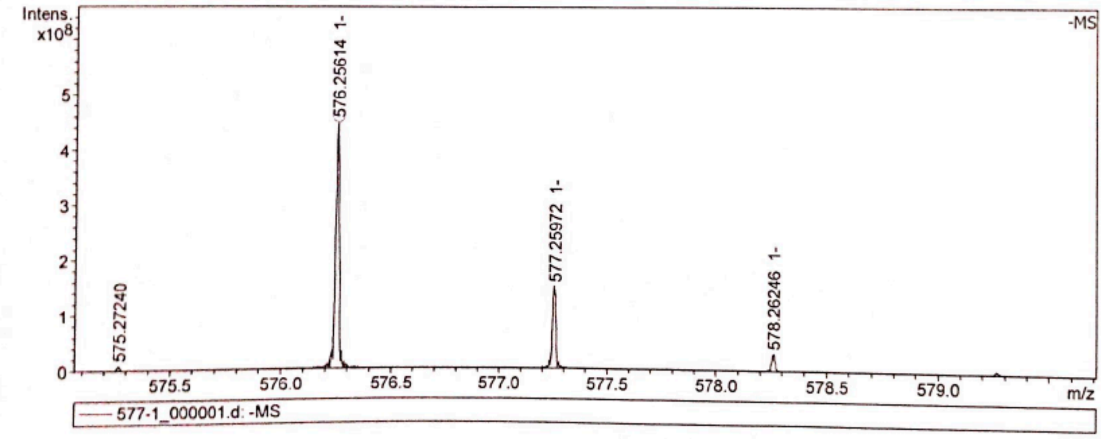

Meas. m/z	#	Ion Formula	Score	m/z	err [ppm]	Mean err [ppm]	mSigma	rdb	e⁻ Conf	N-Rule
187.108827	1	C8H15N2O3	100.00	187.108816	-0.1	-0.3	6.0	2.5	even	ok
304.119054	1	C16H18NO5	100.00	304.119046	0.0	-0.2	8.5	8.5	even	ok
	2	C15H12N8	98.48	304.119041	-0.0	-0.5	9.3	14.0	odd	ok
344.150489	1	C19H22NO5	100.00	344.150346	-0.4	-0.5	6.8	9.5	even	ok
370.129713	1	C20H20NO6	100.00	370.129611	-0.3	-0.4	8.6	11.5	even	ok
	2	C19H14N8O	90.53	370.129606	0.3	-0.7	13.8	17.0	odd	ok
388.140251	1	C20H22NO7	100.00	388.140176	0.2	-0.3	7.7	10.5	even	ok
	2	C19H16N8O2	69.55	388.140170	-0.2	-0.6	11.6	16.0	odd	ok
576.256141	1	C27H32N10O5	100.00	576.256263	0.2	-0.3	2.9	17.0	odd	ok
	2	C28H38N3O10	97.36	576.256268	-0.2	0.0	4.4	11.5	even	ok

Figure S13. HR-ESI-MS of Damxungmacin B (**2**).

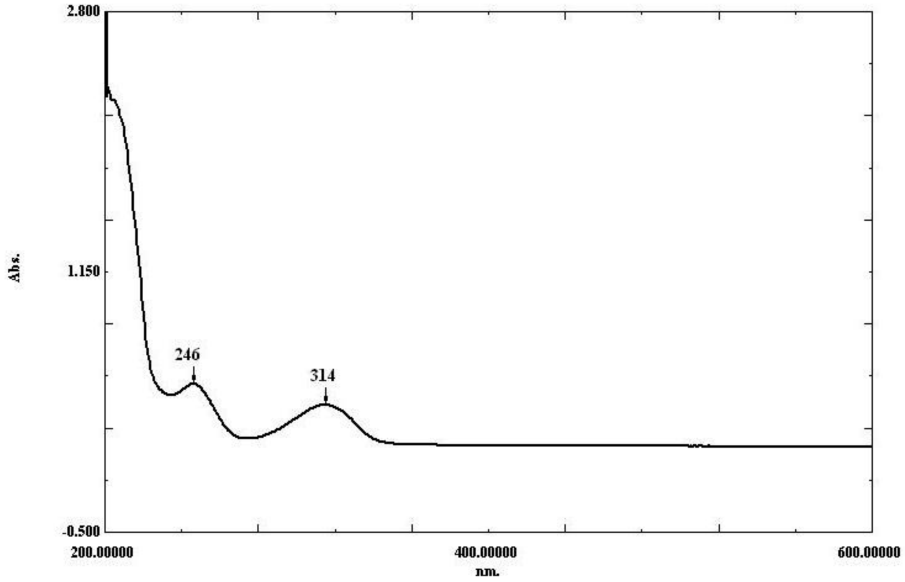

Figure14. UV spectrum of Damxungmacin B (**2**).

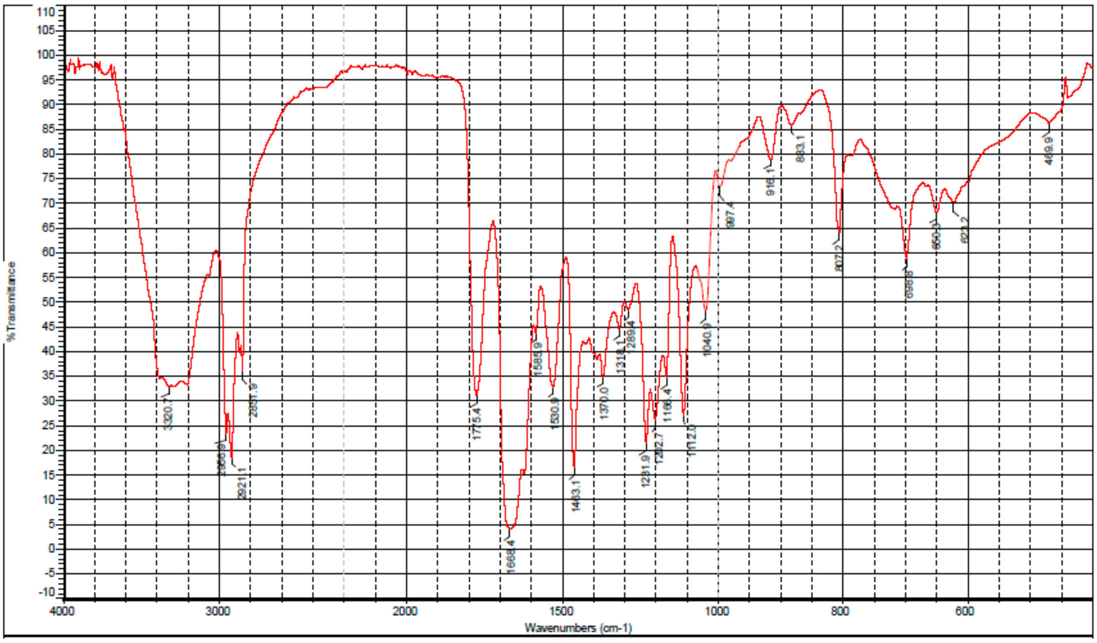

Figure15. IR spectrum of Damxungmacin B (**2**).

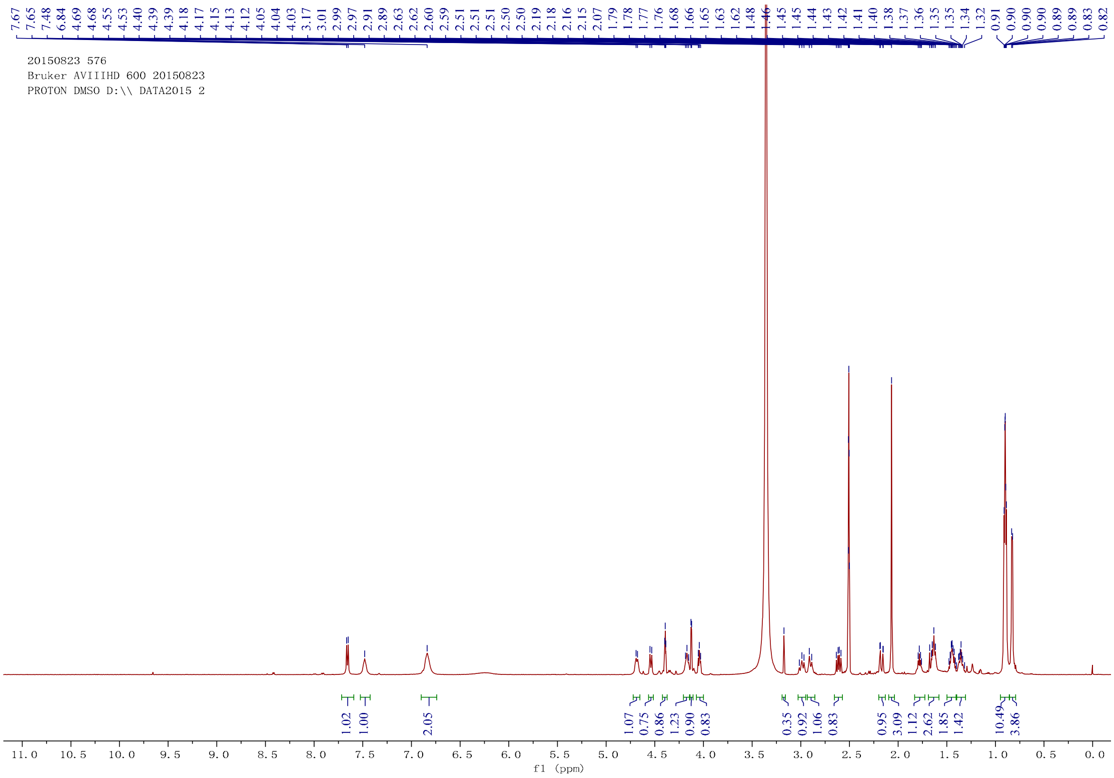

Figure S16. ^{1}H-NMR spectrum of Damxungmacin B (**2**).

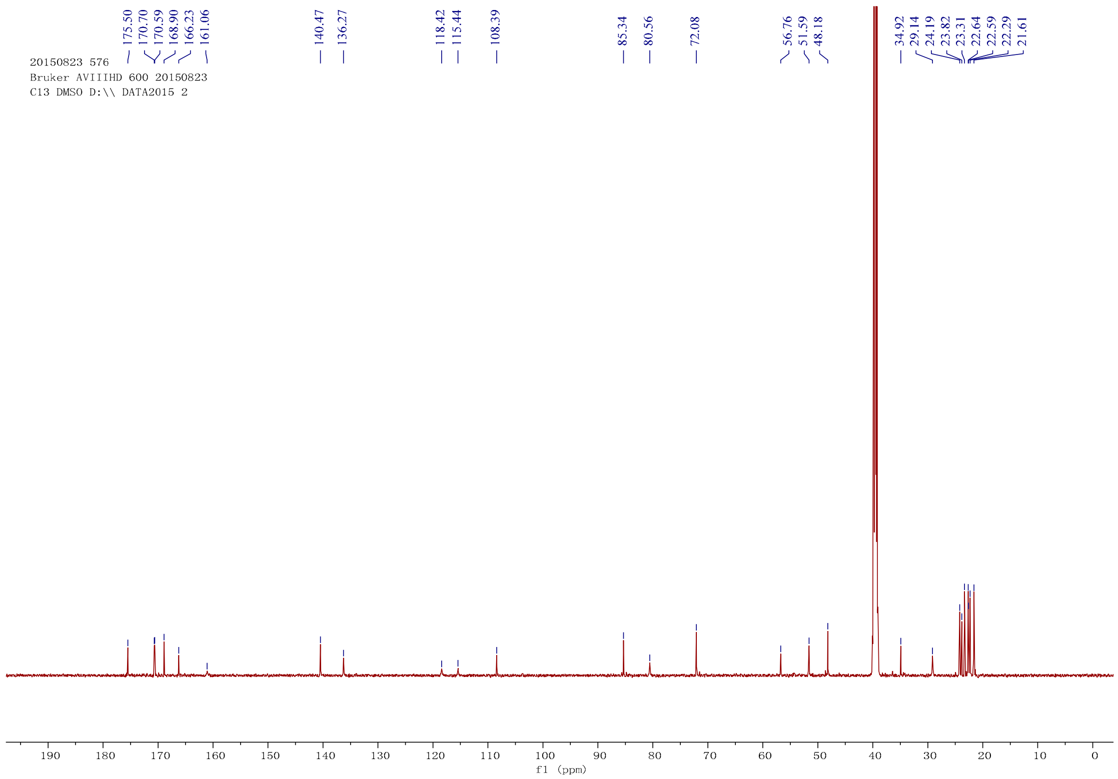

Figure S17. ^{13}C-NMR spectrum of Damxungmacin B (**2**).

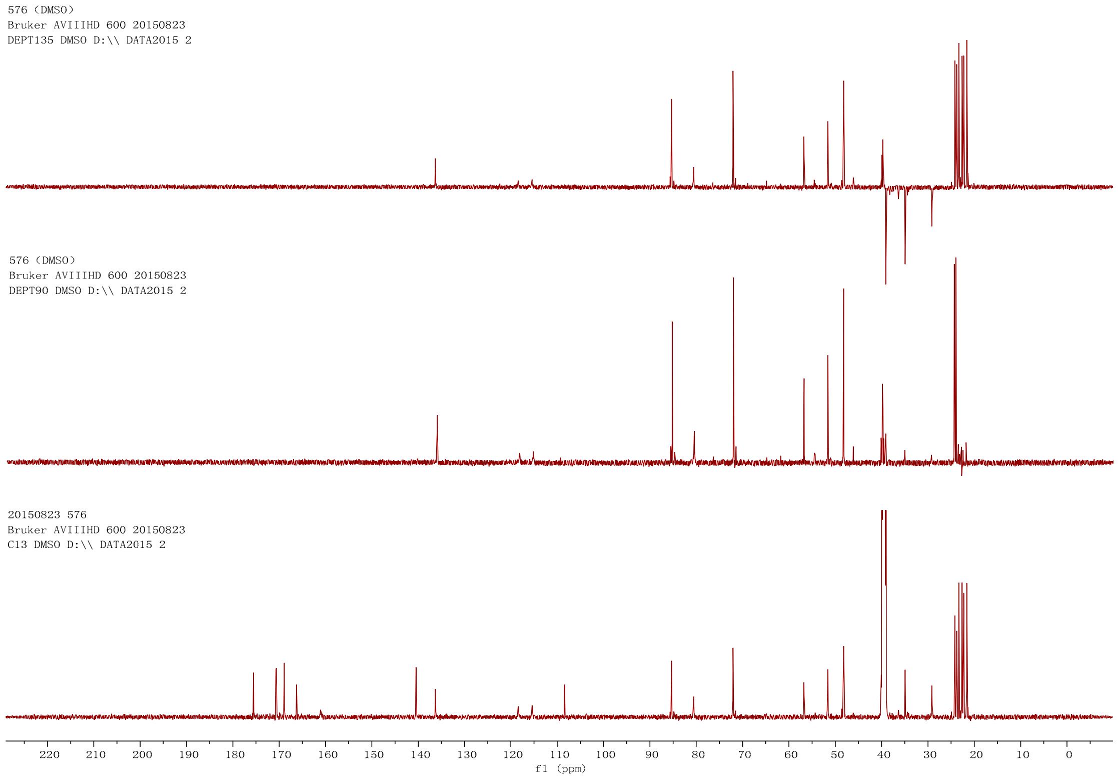

Figure S18. DEPT spectrum of Damxungmacin B (**2**).

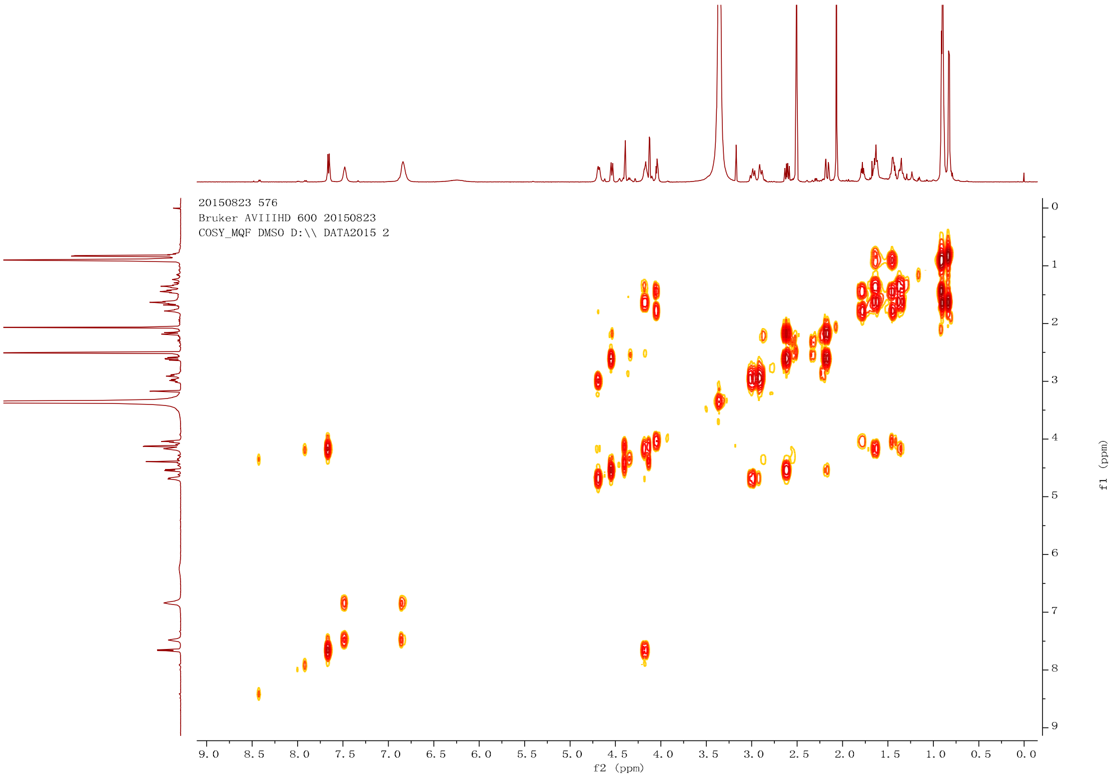

Figure S19. ^{1}H-1HCOSY spectrum of Damxungmacin B (**2**).

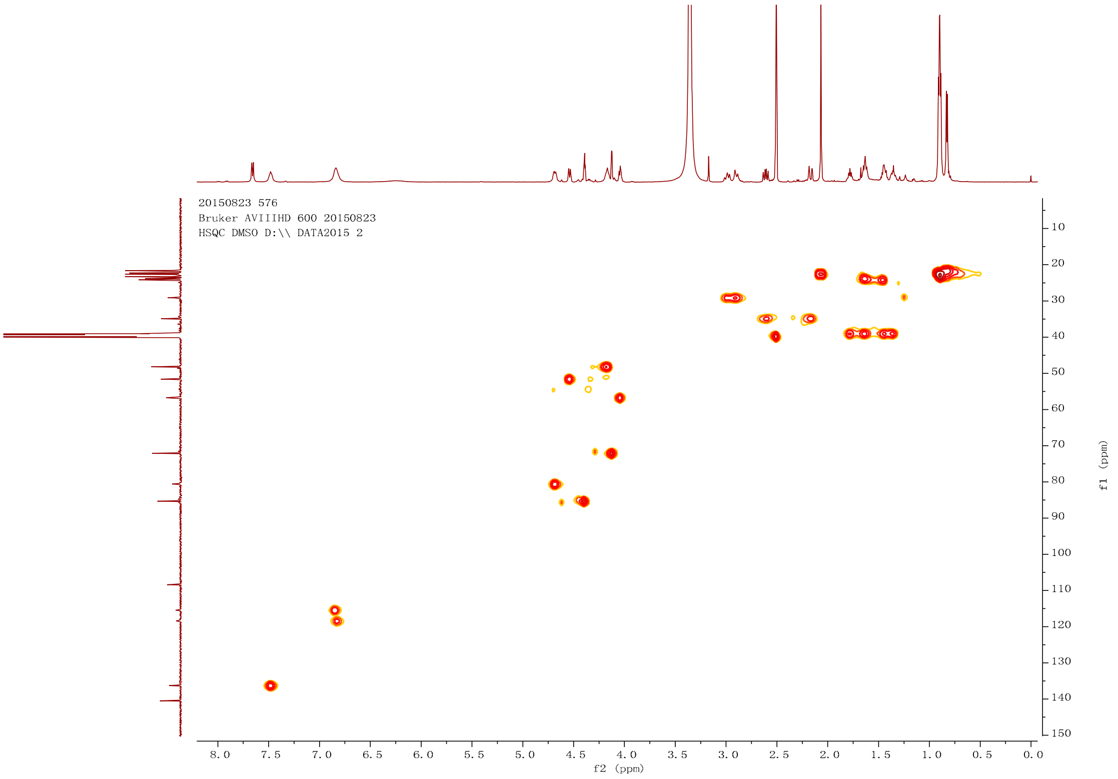

Figure S20. HSQC spectrum of Damxungmacin B (**2**).

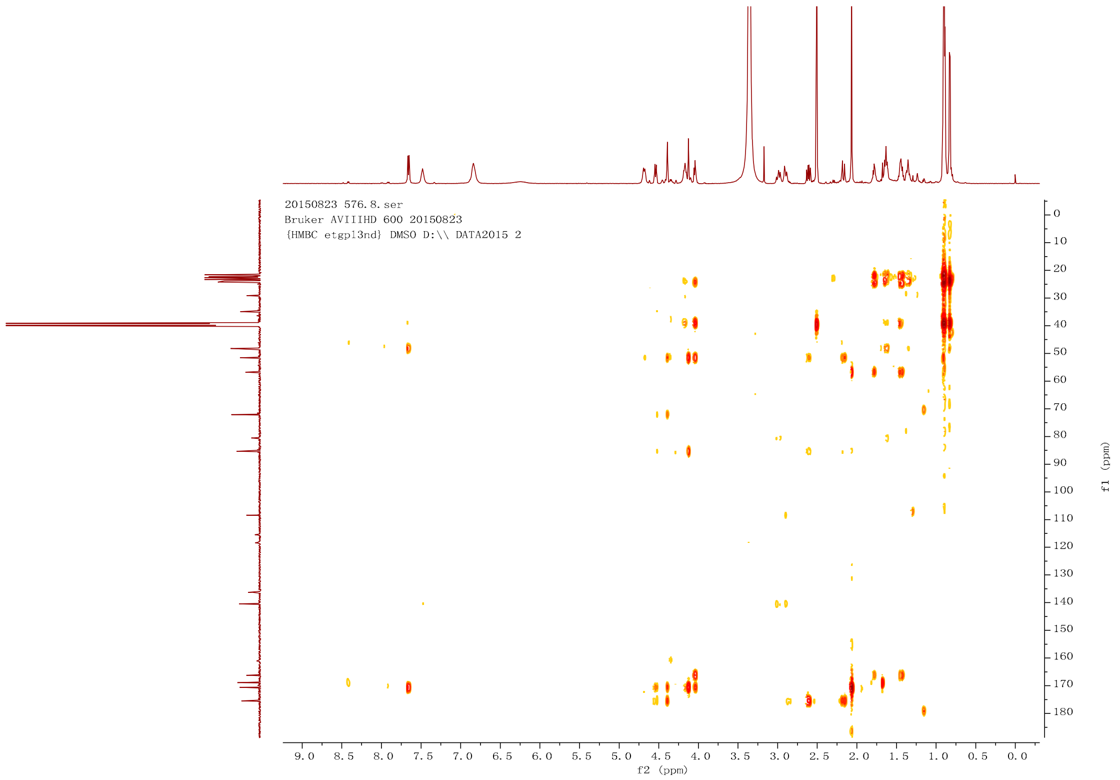

Figure S21. HMBC spectrum of Damxungmacin B (**2**).

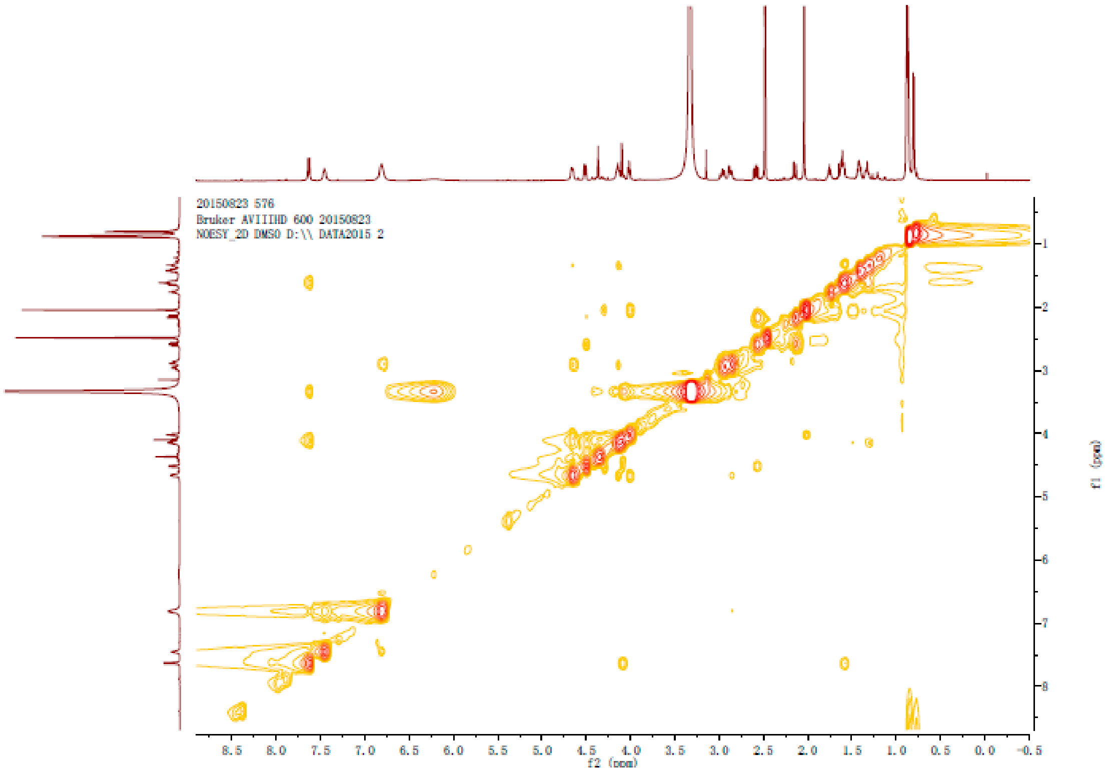

Figure S22. 2D NOESY spectrum of Damxungmacin B (**2**).

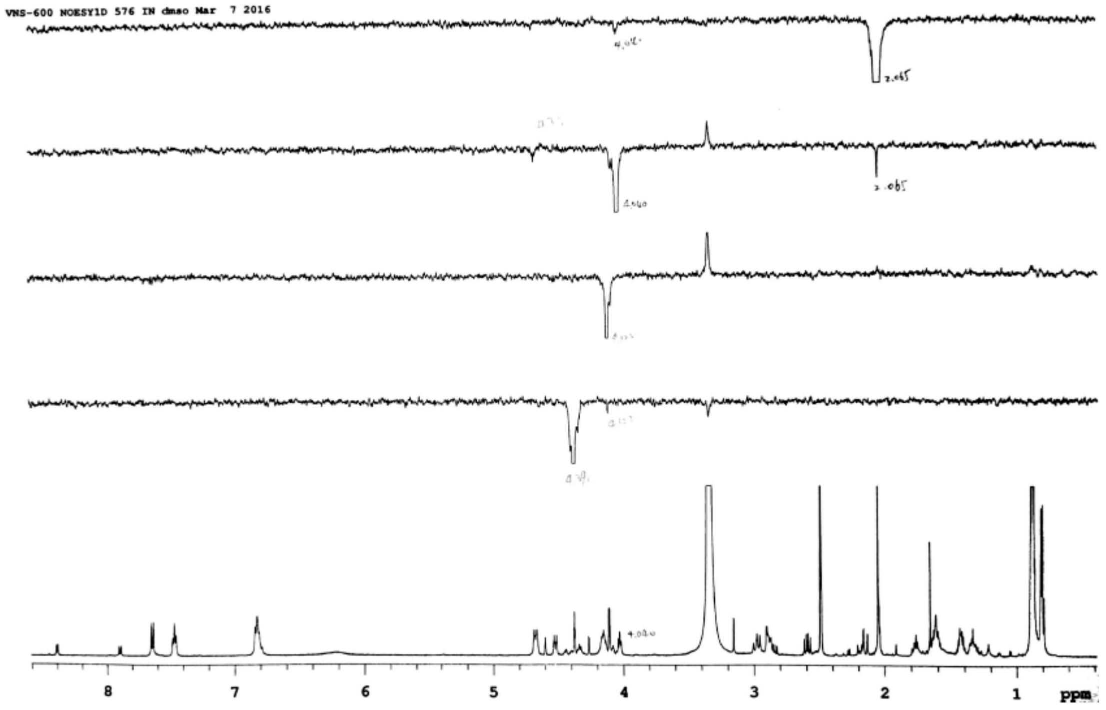

Figure S23. 1D NOESY spectrum of Damxungmacin B (**2**).
